# Observed behaviours of precipitable water vapour and precipitation intensity in response to upper air profiles estimated from surface air temperature

**DOI:** 10.1038/s41598-017-04443-9

**Published:** 2017-07-06

**Authors:** Mikiko Fujita, Tomonori Sato

**Affiliations:** 1 Japan Agency for Marine-Earth Science and Technology, 3173-25 Showa-machi, Kanazawa-ku Yokohama, 236-0001 Japan; 2 Faculty of Environmental Earth Science, Hokkaido University, Kita-10, Nishi-5, Sapporo, 060–0810 Japan

## Abstract

Extremely heavy precipitation affects human society and the natural environment, and its behaviour under a warming climate needs to be elucidated. Recent studies have demonstrated that observed extreme precipitation increases with surface air temperature (SAT) at approximately the Clausius–Clapeyron (CC) rate, suggesting that atmospheric water vapour content can explain the relationship between extreme precipitation and SAT. However, the relationship between atmospheric water vapour content and SAT is poorly understood due to the lack of reliable observations with sufficient spatial and temporal coverage for statistical analyses. Here, we analyse the relationship between atmospheric water vapour content and SAT using precipitable water vapour (PWV) derived from global positioning system satellites. A super-CC rate appears in hourly PWV when the SAT is below 16 °C, whereas the rate decreases at high SAT, which is different from the precipitation-SAT relationship. The effects of upper air temperature and water vapour can consistently explain the super-CC rate of PWV relative to SAT. The difference between moist and dry adiabatic lapse rates increases with SAT, in consequence of more ability to hold water vapour in the free atmosphere under higher SAT conditions; therefore, attainable PWV increases more rapidly than the CC rate as SAT increases.

## Introduction

Many disasters related to extremely heavy precipitation have been reported throughout the worldwide^1^. A likely increase in the intensity and frequency of precipitation extremes is projected by many atmosphere–ocean coupled GCMs with elevated greenhouse gas concentration scenarios^2, 3^. This is an issue for policymakers who must respond to a societal demand to ensure resiliency to disasters caused by heavy precipitation events. The efforts of scientists have been aimed at assessing the degree to which heavy precipitation events would be enhanced in a warming climate. Recently, many precipitation products, such as those from rain gauges^4, 5^, ground-based radar^6^, space-borne precipitation radar^7^, and climate models^2^, have become available for the statistical analyses of heavy precipitation extremes, which requires a high temporal frequency of measurements and a long-term record.

Theoretically based studies have conventionally utilized observed surface air temperature (SAT) for scaling the variation of extreme precipitation intensity, probably because SAT is the most fundamental and reliable meteorological parameter. Relationships between extreme precipitation intensity, which is typically defined as a certain high threshold percentile, and SAT have been investigated in both mid-latitude regions^4–11^ and tropical regions^12, 13^.

**Figure 1:**
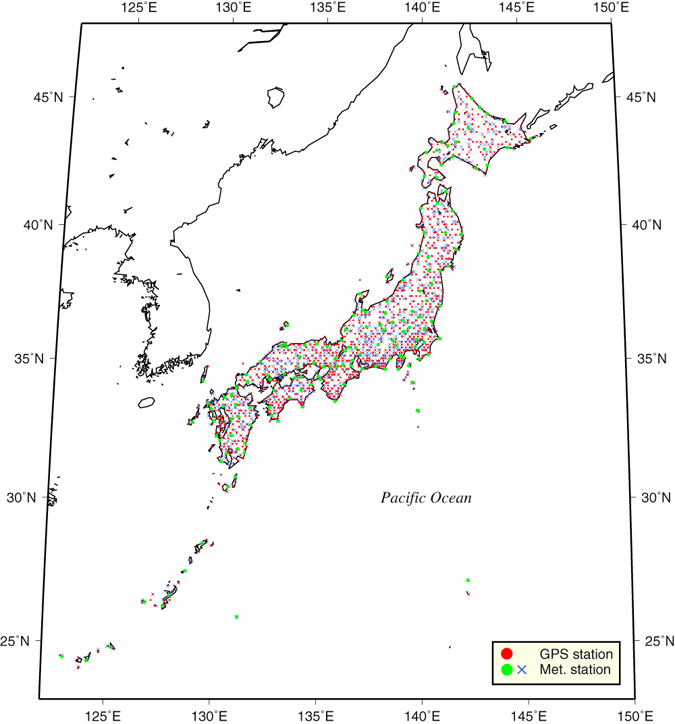
Observational stations. Map of the study area showing GPS and surface meteorological stations in Japan. Red circles indicate GPS stations. Blue crosses and green circles indicate meteorological stations. We use surface pressure, air temperature and relative humidity observed at the green circles and surface air temperature and precipitation observed at the blue crosses. The map was created using GMT Generic Mapping Tools (GMT)^27^ version 5.1.1 available at http://www.soest.hawaii.edu/gmt/.

Recently, as available observational data have accumulated, a consensus has been reached that precipitation intensity can increase more rapidly than the Clausius–Clapeyron (CC) rate if the SAT is above 12–15 °C^4, 9^ or in convective precipitation^6^. However, the physical mechanism that satisfactorily explains this super-CC rate remains controversial. To elucidate the relationship between extreme precipitation and SAT, the tropospheric water vapour content and the vertical profile of air temperature must be considered. Radiosonde observation is a traditional direct measurement of vertical atmospheric structure, but its spatial and temporal coverage is insufficient, especially for capturing short-lived extreme precipitation events that may occur on hourly timescales. In climate models, the atmospheric moisture profile associated with intense precipitation is highly dependent on cumulus and microphysics schemes. Indeed, difficulties in obtaining a dataset of atmospheric water vapour content with high spatial and temporal coverage have been the major impediment to understanding the vertical structure. Here, we use precipitable water vapour (PWV), which is the vertically integrated water vapour mixing ratio, derived from global positioning system (GPS) data archives for the 15 years from 1996 to 2010 to describe a relationship between tropospheric water vapour content and SAT. The GPS-derived PWV data enable sub-hourly estimations of PWV (see Methods) with high spatial density over Japan at ~20 km intervals (Fig. 1).

## Results and Discussion

**Figure 2:**
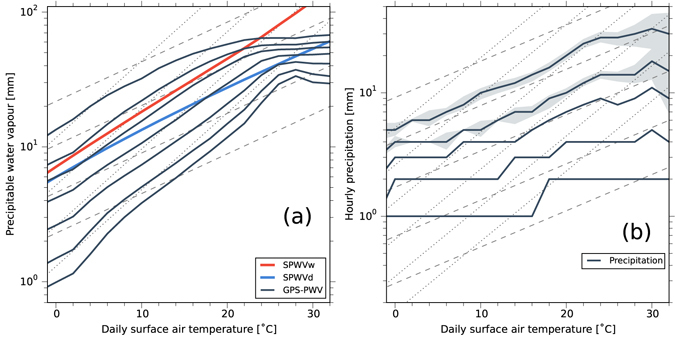
Percentiles of hourly PWV and precipitation on a logarithmic scale as a function of daily surface temperature (SAT). (**a**) Hourly GPS-PWV percentiles. Solid black lines are, from top to bottom, the 99th, 90th, 75th, 50th, 25th, 10th and 5th percentiles of hourly GPS-PWV. Red and blue lines are the estimated SPWV_w_ and SPWV_d_ profiles, respectively. (**b**) Hourly precipitation percentiles. Solid black lines are, from top to bottom, the 99th, 90th, 75th, 50th, and 25th percentiles of hourly precipitation. Dashed and dotted grey lines in both panels are the exponential relations corresponding to one and two times the Clausius–Clapeyron relation, respectively. The shaded area, plotted only for 99th and 90th percentiles, indicates the 90% confidence intervals estimated using the bootstrap method (see Methods).

We analysed all hourly PWV data to determine percentiles for each 2 °C daily SAT bin (Fig. 2a). The 99th percentile PWV increases with super-CC rate, which is larger than the CC rate but less than two times the CC rate, for SAT below 14 °C. Over 14 °C, the extreme PWV increases approximately following CC rate and it become slower. The dependence of extreme PWV (i.e., 99th percentile PWV) on daily SAT differs from that of extreme precipitation^4–11^, which exhibited a super-CC rate in a similar SAT range over the southern part of Japan, where the climatological SAT is high^5, 14^. The 90th percentile PWV also increases with super-CC rate for SAT below 16 °C. Around 18–20 °C, the 90th percentile of PWV increases approximately following the CC rate. For the 50th and 75th percentiles, the super-CC rate appears when the SAT is below 22 °C. Double the CC rate appears when the SAT is between 18 and 24 °C for PWV lower than 25th percentiles, whereas the rate of PWV change becomes slower at all percentiles as SAT increases and tends to decrease at an SAT range higher than 18 °C for 99th percentile and 28 °C for 25th percentile. These features of the PWV-SAT relationship indicate the importance of vertical evolution of the moist layer in the air column, as discussed below.

The percentiles of hourly precipitation at the nearest station to the GPS site were also analysed for each 2 °C daily SAT bin for comparison (Fig. 2b). The precipitation data from observations at 808 stations (Fig. 1) with 1 mm intervals were used, and the percentiles were computed from raw data over 1 mm hr^−1^. The 99th percentile of hourly precipitation increases at a CC-like rate below 16 °C, and it reaches a double-CC rate around 18–22 °C. The percentiles become stable and decrease over 24 °C, although the statistical errors were large. In the 90th, 75th, and 50th percentiles, the hourly precipitation increases at the CC or lower rate. The rates of increase of precipitation are consistent with previous studies of extreme precipitation^4–11^, but there are significant differences in the rates of observed PWV, with super-CC even below 16 °C. Additionally, if daily maximum PWV is used, double-CC rates in lower percentiles are also clearly recognized when the SAT exceeds 18 °C (Supplementary Fig. 1). Key questions that are raised include why does PWV increase in super-CC rate with SAT and how does the maximum water vapour capacity in the air column (saturated precipitable water vapour: SPWV) vary with SAT?

In the CC equation, the water vapour capacity of a given volume of air is constrained by the air temperature at that height, not necessarily by the SAT. To interpret the CC rate in observed PWV, we compare it with theoretically obtained SPWV indices, SPWV_d_ and SPWV_w_, by assuming two tropospheric temperature profiles corresponding to the dry and wet adiabatic lapse rates (Γ_d_ and Γ_m_), respectively, for an air parcel at ground level with a temperature equal to the SAT (see Methods). The rate of increase in SPWV_d_ is close to the CC rate of 7% °C^–1^, confirming that the CC relationship holds between the theoretically derived attainable PWV and SAT for sub-saturation conditions (blue line in Fig. 2a). By contrast, SPWV_w_ increases more rapidly than the CC rate, reaching 11–12% °C^–1^ (red line in Fig. 2a). This pattern is due to the increasing amount of latent heat energy released per unit SAT rise, similar to the relationship between potential temperature and saturated equivalent potential temperature as their difference becomes larger with increasing air temperature. The GPS-PWV lies above SPWV_d_ at the 75th, 90th, and 99th percentiles, indicating that there is more water vapour than in a standard sub-saturation atmosphere. Furthermore, the 90th and 99th percentiles PWV are above the SPWV_w_ for SAT below 22 °C. These findings require a further analysis of the vertical distribution of water vapour.

**Figure 3:**
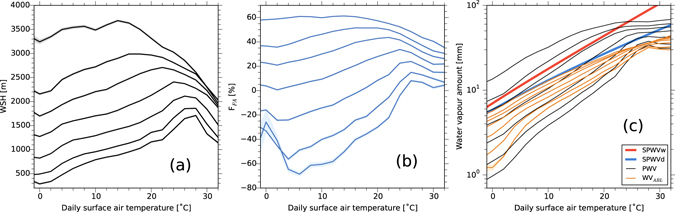
Percentiles of water vapour parameters as a function of SAT. (**a**) WSH. (**b**) Percentage of water vapour amount in the free atmosphere (F_FA_). (**c**) Water vapour amount in the boundary layer (WV_ABL_; orange), GPS-PWV (black), SPWV_w_ (bold red) and SPWV_d_ (bold blue). Multiple lines of WSH, F_FA_, and WV_ABL_ represent the values when the 99th, 90th, 75th, 50th, 25th, 10th and 5th percentiles values of GPS-PWV (from top to bottom in the figures) are observed. In panel (**c**), the GPS-PWV percentiles are the same as in Fig. 2a except for the number of stations used. The shaded area indicates the 90% confidence intervals (see Methods). Note the vertical log scale in (**c**).

To discuss the vertical distribution of water vapour, the observed GPS-PWV is separated into two parts: water vapour in the boundary layer that is assumed to be constrained by SAT and water vapour in the free atmosphere that reflects deep convective processes. To determine the typical boundary layer height, we adopt the water vapour scale height^15^ (WSH; see Methods) as an index representing how deeply the near-surface atmosphere is mixed by thermal turbulent eddies. In Fig. 3a, estimated WSHs are plotted for each percentile of GPS-PWV. The WSH increases with PWV percentiles: the maximum WSH for the 99th percentile reaches 3,500 m at an SAT of 14 °C, whereas the 25th percentile has a maximum WSH of 2,100 m at 26 °C. The maximum WSH tends to appear at higher SAT as the percentile value decreases. For WSHs below the 25th percentile, WSH increases with SAT until 26 °C, whereas it decreases at SAT over 28 °C. At other percentiles, WSH also increases when SAT is below 14–24 °C with a lower rate of increase than at lower percentiles. The WSH decreases with SAT at SAT over 14 °C for 99th percentile and over 24 °C for 50th percentile at a rate similar to that at the lower percentiles.

Based on the WSH, the percentage of water vapour in the free atmosphere (above the WSH) relative to PWV was analysed to explain the super-CC rate in observed PWV. If the saturated atmosphere in the boundary layer is considered, the maximum amount of water vapour within the WSH (WV_ABL_; see Methods) is determined by the vertical integral of the saturated water vapour mixing ratio for the temperature profile assuming SAT and Γ_d_. The WV_ABL_ (orange lines in Fig. 3c) in the higher percentile is very similar to SPWV_d_ (blue line in Fig. 3c) and the CC rate (see Fig. 2a). The amount of water vapour in the free atmosphere (WV_FA_) is calculated as the difference between PWV and WV_ABL_. The percentage fraction of WV_FA_ relative to PWV (F_FA_; see Methods) is stably high with positive values at high percentiles (Fig. 3b). Indeed, at the 99th percentile, approximately 50% of PWV is in the free atmosphere when SAT is below 20 °C, indicating a deep moist layer. For lower percentiles, the F_FA_ is negative for low SAT but becomes positive as SAT increases. The change in F_FA_ from negative to positive with increasing SAT suggests the vertical expansion of the moist layer from the near surface into the free atmosphere.

As upward moisture transport is vital for moist convection, the F_FA_ is high for high percentiles, as shown in Fig. 3b. Furthermore, for high SAT, convective precipitation is likely to dominate, which also contributes to the vertical expansion of the moist layer by pushing up lower-level water vapour^6^. For the 99th percentile of precipitation (Fig. 2b), the rate of increase with SAT around 16 °C exceeds the CC rate, reaching the double-CC rate. At SAT around 16 °C, the water vapour in the free atmosphere significantly increases even in the 25th percentile (Fig. 3b). Additionally, the PWV during heavy precipitation events shows a higher increasing rate with SAT (Supplementary Fig. 2).

These results also support the hypothesis that at higher SAT, vertical water vapour transport in convective precipitation systems penetrates into the free atmosphere, charging more water vapour above the boundary layer in addition to the increased water vapour holdings in the boundary layer constrained by SAT. As the surface precipitation intensity depends on both cloud microphysical processes and mesoscale circulations, the precipitation intensity does not always have a linear relationship with PWV. However, the PWV analysis indicates that vertical transport of water vapour is vigorous under high SAT that allows enhanced water vapour in the free atmosphere as condensation heating warms the air temperature. The vertical distributions of air temperature and water vapour mixing ratio are crucial for describing the behaviour of convective systems under different SAT conditions^16^.

For an SAT higher than 16 °C, the rate of increase in the 99th percentile GPS-PWV is reduced (Fig. 2a), which is associated with the reduction of WSH (Fig. 3a). High SAT tends to appear in boreal summer, when there is a quasi-stationary anticyclone around the Western North Pacific as part of the subtropical high belt. The surface divergent flow under the high-pressure system reduces PWV because the descending airflow transports low-moisture air downward, which prevents the wetter surface layer from developing vertically. On hot summer days with an SAT higher than approximately 20 °C, the stable atmospheric profile is unfavourable for developing convective precipitation. A similar decrease of precipitation at high SAT was observed in other regions when the dominant synoptic circulation in a certain SAT range did not attract intense precipitation^12, 17^. In addition, the PWV is less sensitive to SAT during winter, as the near-surface atmospheric layer tends to be capped by an inversion layer. The study area is influenced by a continental air mass during the winter monsoon that efficiently reduces the GPS-PWV. Therefore, the rapid increase in low-percentile PWV with SAT (Fig. 2a) is caused mainly by a skewed probability distribution with a long tail on the low-PWV side, which likely reflects the dominant synoptic patterns in the study area. These features show that synoptic circulation change is as important as SAT because it can alter the probability distribution of precipitation intensity, as indicated by future projected changes in mean precipitation that will involve decreases in many regions of the world despite increasing SAT^18^.

In this study, a theoretical analysis has revealed that the rate of SPWV increase with SAT becomes logarithmically steeper at high SAT (red line in Fig. 2) because of the increasing contribution of water vapour condensation heating that warms the upper air and thus increases water vapour content nonlinearly under moist convection. A quantitative evaluation of the source of increased PWV with SAT, i.e., how much of a contribution is derived from horizontal moisture convergence under different seasons or geographical backgrounds, would be an interesting topic for future studies. Although this study is just one example in a humid climate zone, the fundamental idea is applicable to other areas. The effect of latent heating in a cloud system on the vertical temperature profile is a key consideration to answer the general question “How much will PWV increase, and how strong will extreme precipitation be, in a future climate?”.

## Methods

### GPS precipitable water vapour

The GPS raw signal data from 1,219 stations provided by the GNSS Earth Observation NETwork system (GEONET) of the Geospatial Information Authority of Japan (Fig. 1) were used for the calculation of PWV over the period 1996–2010. The PWV was calculated from zenith tropospheric delay (ZTD), which is a measure of the communication time lag between GPS satellites and the GPS receiver. The moisture component of ZTD was converted to PWV by removing the effect of dry atmosphere estimated from the surface air pressure^19^ and SAT^20^ at meteorological stations near the GPS station. The ZTD was computed by the GPS processing software RTNet^21, 22^, and the coordinates of the GPS sites were estimated monthly to avoid any un-modelled geodetic error^23^ or site-specific biases, which can affect the observed vertical depth of the atmosphere. Here, the Saastamoinen troposphere model^24^ and the global mapping function^25^ were used. The observation cut-off angle was 10°, and the data-sampling interval was 30 seconds^26^. Finally, the estimated GPS-PWV was averaged hourly. The GPS-PWV represents the mean PWV in the atmosphere over an inverted conical shape with a base radius of approximately 30 km. The accuracy of the GPS-PWV was verified by a comparison with PWV derived from a direct humidity profile observed by radiosonde, and it was shown to be sufficiently high^26^.

In the present study, the GPW-PWV data were rejected if the surface pressure was below 980 hPa to avoid the observational errors that may occur if the horizontal atmospheric gradient is very large, such as around typhoons.

### Statistical tests

The percentiles of PWV and precipitation were computed from the raw data. The 90% confidence intervals of the percentiles were determined using the bootstrap method. After 3,000 estimations of percentile values by using random subsamples, 90% confidence intervals were determined based on the assumption that 3,000 estimated values follow a normal distribution. The number of subsamples was set to 10% of all data in each SAT bin. The estimated confidence intervals of PWV are very small and difficult to visually identify in the figures.

### Calculation of theoretical saturated precipitable water vapour

The theoretical saturated water vapour (SPWV) was estimated as follows. First, vertical air temperature profiles were estimated at 100 m intervals. An air parcel with temperature equal to SAT and pressure of 1,013 hPa at the ground was lifted to 10 km by applying the dry or wet adiabatic lapse rate. Two SPWV values, SPWV_d_ and SPWV_w_, were then obtained for each of the two air temperature profiles by calculating the vertical integrals of the saturated mixing ratio of water vapour assuming the hydrostatic balance. Here, SPWV_d_ and SPWV_w_ represent the attainable PWV corresponding to dry and wet adiabatic lapse rates, respectively, and hence are expressed as functions of SAT. We repeated these steps for SAT varying from −2 to 32 °C at 2 °C intervals. The dry adiabatic lapse rate (Γ_d_) is a constant (0.98 K 100 m^−1^), whereas the wet adiabatic lapse rate (Γ_w_) varies from approximately 0.35 to 0.98 K 100 m^−1^. The wet adiabatic lapse rate depends on the saturated water vapour mixing ratio as

1 $${{\rm{\Gamma }}}_{{\rm{w}}}={{\rm{\Gamma }}}_{{\rm{d}}}/(1+\frac{L}{{c}_{p}}\frac{d{q}_{{\rm{s}}{\rm{a}}{\rm{t}}}}{dT})$$

where *L* is the latent heat of condensation of water (2,501 kJ kg^−1^), *c*_*p*_ is the specific heat of dry air at constant pressure (1,003.5 J kg^−1^ K^−1^), *q*_sat_ is the saturated water vapour mixing ratio, and *T* is air temperature.

### Estimation of boundary layer height and water vapour amount in and above the boundary layer

The scale height of water vapour WSH was derived as^15^.

2 $${\rm{W}}{\rm{S}}{\rm{H}}={\rm{P}}{\rm{W}}{\rm{V}}/({\rho }_{d}{q}_{{\rm{s}}{\rm{f}}{\rm{c}}})$$

where *q*_sfc_ is the water vapour mixing ratio at the surface and *ρ*_*d*_ is the density of dry air (1.2 kg m^−3^) at the surface. For WSH, we used only 321 GPS stations within 20 km of the meteorological stations where observations of relative humidity and air pressure, required for calculating *q*_sfc_, are routinely performed with hourly interval (green circles in Fig. 1). The WSHs (Fig. 3a, black line) corresponding to each PWV percentile were calculated as the arithmetic mean of hourly interval WSHs for the events when the PWV value was within the 90% confidence interval for each percentile (Fig. 2a, shading).

The amount of water vapour in the boundary layer (WV_ABL_) was calculated based on the vertical integral of the saturated water vapour mixing ratio (*q*_sat_) between the ground surface and WSH height. Here, we considered the vertical distribution of *q*_sat_ assuming SAT and Γ_d_ similarly to the calculation of SPWV_d_. For this calculation, we also used air pressure observed at ground surface level and that at WSH level assuming the hydrostatic balance. As the PWV can be divided into two components—water vapour in the boundary layer (WV_ABL_) and water vapour in the free atmosphere (WV_FA_)—the following can be derived:

3 $${{\rm{WV}}}_{{\rm{FA}}}={\rm{PWV}}-{{\rm{WV}}}_{{\rm{ABL}}}$$

The fraction of water vapour in the free atmosphere (F_FA_) is defined as the percentage of WV_FA_ relative to the PWV, i.e., F_FA_ = WV_FA_/PWV * 100. The negative F_FA_ can occur at the low percentiles of PWV because saturated mixing ratio was used in estimating WV_ABL_. Therefore, the negative F_FA_ for the low PWV percentiles means that water vapour holding capacity in the boundary layer is larger than PWV (WV_ABL_ > PWV), whereas the positive F_FA_ means that the PWV is sufficiently high to exceed the holding capacity of water vapour in the boundary layer (WV_ABL_ < PWV), suggesting that the moist layer develops vertically beyond WSH.

## Electronic supplementary material

Supplementary Information
