# Supplementary material for: Observed behaviours of precipitable water vapour and precipitation intensity in response to upper air profiles estimated from surface air temperature
Source: Sci Rep. 2017 Jul 6;7:4233. doi: 10.1038/s41598-017-04443-9 (PMC5500547; doi:10.1038/s41598-017-04443-9)
Supplement: Supplementary file 1 — Supplementary Information [file 41598_2017_4443_MOESM1_ESM.pdf]

Observed behaviours of precipitable water vapour and precipitation intensity in response to upper air profiles estimated from surface air temperature

Supplementary Information

Mikiko Fujita and Tomonori Sato

Supplementary Figures

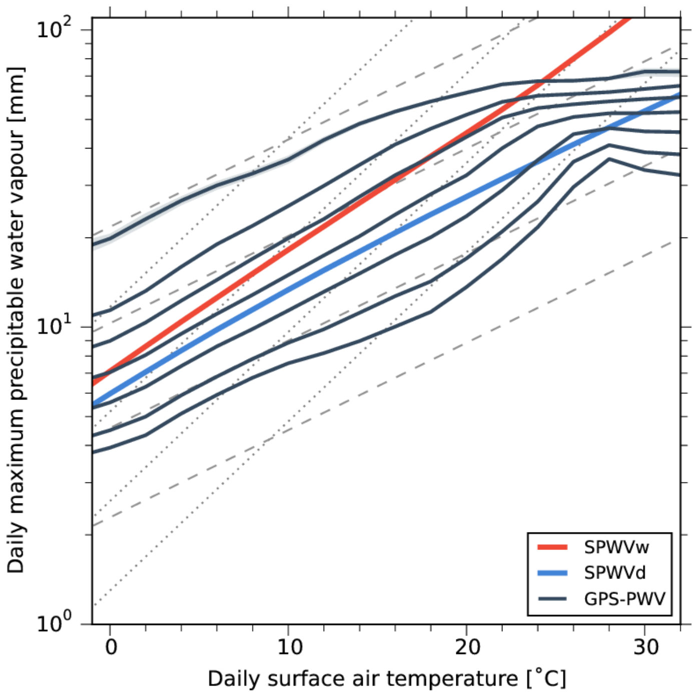

Figure S1 | Percentiles of daily maximum of hourly PWV on a logarithmic scale as a function of daily surface temperature (SAT). Solid black lines are, from top to bottom, the 99th, 90th, 75th, 50th, 25th, 10th and 5th percentiles of daily maximum of hourly GPS-PWV. Red and blue lines are the estimated $SPWV_w$ and $SPWV_d$ profiles, respectively. Dashed and dotted grey lines are the exponential relations corresponding to one and two times the Clausius–Clapeyron relation, respectively. The shade area, plotted only for 99th percentiles, indicate 90 % confidence intervals estimated by the bootstrap method (see Methods).

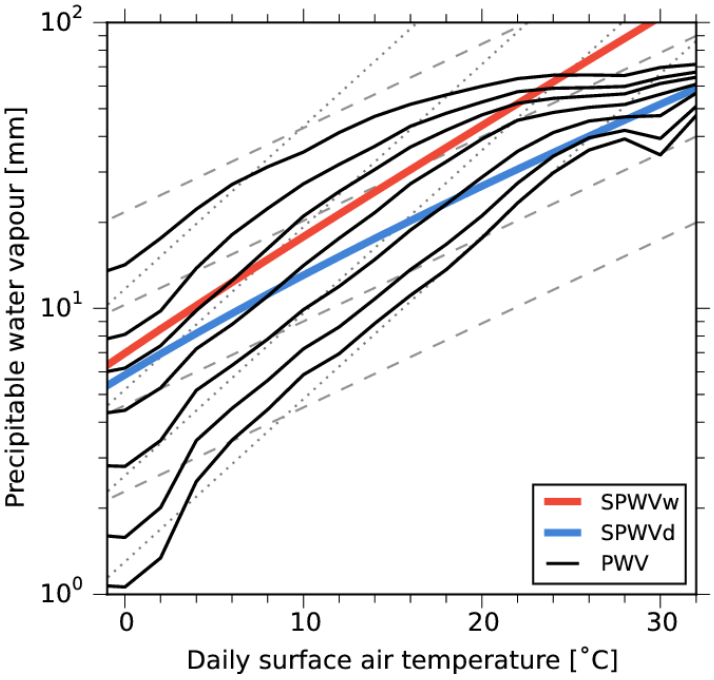

Figure S2 | Percentiles of hourly PWV before and after extreme precipitation on a logarithmic scale as a function of daily surface temperature (SAT). Solid black lines are, from top to bottom, the 99th, 90th, 75th, 50th, 25th, 10th and 5th percentiles of hourly GPS-PWV. These GPS-PWV percentiles were calculated from the data when the extreme precipitation over 95th percentile in the each SAT bin occurred in the nearest meteorological stations (blue crosses in Fig.1) during 6 hours before and after. If multiple extreme hourly precipitation events occur within the 12 hours, we regard them as one event to avoid duplicative count of GPS-PWV data. Red and blue lines are the estimated $SPWV_w$ and $SPWV_d$ profiles, respectively. Dashed and dotted grey lines are the exponential relations corresponding to one and

two times the Clausius–Clapeyron relation, respectively. The shade area, plotted only for 99th percentiles, indicate 90 % confidence intervals estimated by the bootstrap method (see Methods).
